# Recent Contributions to the Literature of Comparative Medicine

**Published:** 1881-01

**Authors:** Thomas E. Satterthwaite


// THE JOURNAL

OF

COMPARATIVE MEDICINE AND SURGERY.

VOL. II. JANUARY, 1881. No. 1.

### ORIGINAL COMMUNICATIONS.

### ART. I.—RECENT CONTRIBUTIONS TO THE LITERATURE OF COMPARATIVE MEDICINE.

### PART II.

### THE CYSTICERCUS CELLULOSÆ OR MEASLES DISEASE OF ANIMALS AND MEN.

THIS disease has been recognized from very early times; indeed, it is said that Aristophanes described it in the hog, and Aristotle was also aware of it. Our present knowledge of the way in which it effects an entrance into the body, and its life history, are due largely to the investigations of Küchenmeister, Haubner, Heller, and some others. This cysticercus, according to the latter authority, is the larval or immature form of the common tape-worm—the taenia solium, that is said to occur only in the small intestine of man. The cyst which lodges the parasite is seldom larger than a bean, and contains a clear fluid. The little body, of a white or yellowish color, is enclosed in a pyriform sac, and depends from the wall of the vesicle. The head is surmounted by four suckers and a double row of hooks, each one of which rests in a little pouch-like depression. Occasionally the cysticerci are found free, that is, they have no capsule—as in the ventricles of the brain. In such instances, they are more or less clustered together and united by connective tissue filaments with the floors of the ventricles, or trunks of nerves and vessels. When the embryonic cysticercus first locates himself in the tissue, he has only six hooks; these are soon thrown off, and then the gradual development of the permanent larval form begins. This phase of life history is completed in about two-and-a-half months, while the total duration of life varies from three to six years. The final condition is often that of a little calcified mass, in which the hooklets may be artificially dissolved out by any solvent of lime salts, such as muriatic acid. At such an advanced period it is plain that there is no danger of infection. The measle is said to have been found in the ape, the bear, dog, rat, and deer. It may occur in any part of the body, but is chiefly lodged in the intermuscular connective tissue. The symptoms will often be insignificant, and yet when the brain or eye is involved, they may be very violent. After the larval process is entirely completed, the morbid manifestations usually abate.

It is difficult to make a diagnosis of the disease, except when it is accessible to the knife, as in the skin, or may be detected by the opthalmoscope. Successful treatment, therefore, is only possible in a limited number of instances. But even acting on the supposition that the system at large is positively known to be infected, we are not possessed of any remedy that will destroy the parasites without doing a probable harm to the unhappy bearer. Prophylaxis, accordingly, is the object to which attention should be mainly given. Individuals may be diseased in two ways:

1. By self-infection. A person having a tape-worm may regurgitate a mature joint or segment into his stomach. The digestive juices dissolve the joint, and the six-hooked embryo is ready to start out on his journey, which he does very often by directing his path towards the liver. The precise avenues he takes, whether the blood-paths or lymphatics, is unknown. He may possibly pierce directly through the tissues.

2. The mature eggs from another person, after expulsion from the bowel, may in some way gain access to the body, possibly with the drink. Hogs are remotely the cause, because they lodge the measles, which, when swallowed by the human subject, under the form of raw or under-done pork, or even smoked or pickled meat, will produce the tape-worm, and this, in turn, the measles in man, as first described. Eradication of the disease from swine is therefore a matter of the very first importance, and should occupy the attention of all our public sanitary boards.

The following recent case is given by Heller, as contributed by Dr. G. Merkel. A working girl, twenty-three years old, of previous good health, began to complain of headache. After a violent attack, where maniacal symptoms were developed, she was ordered to the hospital, but died on the way. The post-mortem examination revealed extreme anæmia of the brain, with a slight degree of chronic internal hydrocephalus; on the corpora quadragemina there was a tumor, the size of a small hazel nut, loosely united to the membranes. It consisted of five or six very delicate, transparent little cysts, the diameters of small peas, and clustered together like grapes, each with a terminal vesicle the size of half a bean. One of the little cysts exhibited a minute white point in the centre, the size of a pin-head. Under the microscope it was seen to be the head of a cysticercus, with twenty-nine adherent hooklets and two loose ones.

The same author also mentions an instance in which a post-mortem examination revealed an infiltrated spot in the lung; in the centre was a cavity, the size of a barley-corn, distended with a transparent vesicle, which proved to be a cysticercus, in which there was a rudimentary development of the head. No other larvæ were found in the body.

In view of the fact that the disease is communicated to swine through human excrement, and that the hog returns the disease in the altered form of the tape-worm to the original donor, it is plain that two things are necessary, from a prophylactic point of view. Pork should be carefully inspected with a view to rejecting all "measly" meat, and hogs should never be allowed any possible access to human ordure, which is a matter of every-day occurrence in our towns and villages. Severe penalties should attach to the neglect of this latter important sanitary regulation.

It is interesting to note that numerous larvæ may be present, and yet not produce symptoms of any sort. Most frequently, however, they give rise to slight or more severe symptoms; indeed, under certain circumstances a single one is capable of producing death.

The cysticerci at times undergo contractions, and it is thought possible that marked cerebral phenomena may be due to this peculiar movement which belongs to them. Hitzig managed to induce real epileptic attacks in animals (dogs), by introducing particles of degenerate material into the cortical portion of the brain. He inferred that the action of the cysticerci might be similar in kind.

Dr. C. S. Turnbull, of Philadelphia, discovered one of these parasites in the eye of a patient, and has given the following history of the case: A patient (aet. 55) was attacked with pain in an eye that had been the seat of cataract for about fifteen years. The pains increased in intensity, and were paroxysmal, the feeling being described as if there were "strokes of a hammer inside of the eye." There was also vascular engorgement and profuse lachrymation. On opthalmoscopic examination, a sac was seen in the posterior chamber. It was united to the ciliary margin. The next day the diagnosis was assured, from the fact that the parasite was seen detaching itself. On the third day he was floating free in the aqueous humor. The appearance of the cystitercus was astonishingly like a balloon, of which the sac was the gas receptacle, and the head the basket. In moving about, he withdrew himself, from time to time, into the balloon, and then suddenly threw himself out. He was removed on the ninth day by Graefe's operation for cataract.

The following case has already been alluded to as an example of the possible difficulties attending diagnosis in a case of hydrophobia: A woman, aged fifty-two, was bitten in the face by a dog. All efforts at swallowing were attended with severe and painful spasms. A white, sticky saliva flowed from her mouth; blowing upon the face also produced reflex spasms. This case, which was diagnosticated as canine rabies, was regarded by Mr. Dolan, who reports it, as due to cysticerci, for at the post-mortem examination there was found at the base of the brain a pyriform cyst, the size of a large nut, "which penetrated into the fissure of Silvius." The tumor was filled with cysticerci.

---

HITZIG.—Abhandl. phys. u. path. Inhaltes. Berlin, 1874.

KIPP—Trans. of Internat. Med. Congress. 1876.

HELLER.—Ziemssen's Handbuch, III., Bd. 2 Auflage, 1876.

SMITH (R. S.)—Cysticercus cellulosæ found in the lateral ventricle of the brain. Tr. Br. Med.-Chir. Soc., 1878, i., 68-70, 1 pl.

COBBOLD.—Entozoa. London, 1879.

DOLAN.—Practitioner. July, 1879.

MEDIN (O).—Ett fall f. cysticercus cellulosæ cerebri. Hygeia. Stockholm, 1879, XL1X. 226.

MARCHAND (F.)—Cysticercus of the brain. Arch. f. Path. Anat. Berlin, 1879, 7. F., V. 104-112.

CALHOUN.—Med. and Surg. Reporter. Philadelphia, June, 1879.

TURNBULL (C. S.)—On cysticercus cellulosæ. Tr. Med. Soc. of Penn. Philadelphia, 1879, xii., pt. 2 p, 674, 681.

FRANK (E.)—Ein fall von cysticerken im Herzen und Gehirne. Allg. Wien, Med. Ztg., 1879, xxiv., 376.

FISCHER (C.)—Cysticercus cellulosæ in musc. biceps. Berlin Klin Woch, 1879, xvi., 730.

SCHIFF (E.)—Ein fall von cysticercus cellulosæ. Viertljhr. f., Derm., Wien., 1879, vi, 275-278.

SOLA (E. G.)—Exámen microscópic del cysticercus cellulosae en lar carne del cerdo. Siglo Med. Madrid, 1879, xxiv., 241-244.

MACHIAVELLI (P.)—Cisticerchi nel cervello. Gazz. Med. Ital. lomb. Milano, 1879, 8 s. i., 253.

FRANK (E.)—Ein Fall von Cysticerken im Herzen und Gehirne. Allg. Wien., Med. Ztg., 1879, xxiv., 376.

MUNKENBECK (L.)—Cysticercus cellulosæ und demselben ähnliche Gebilde. Wochenschr. f. Theierh. u. Viehzucht. Augsburg, 1880, xxiv., 87.

THE ECCHINOCOCCUS OR "HYDATID" DISEASE.

The ecchinococcus is now generally known to be the larval or immature form of the tænia ecchinococcus, a small cestode or tape-worm that infests the dog. Its length is only about one-eighth of an inch, and it is usually lodged in the upper part of the small intestine. It differs chiefly from the ordinary tape-worm in its smaller size and the limited number of joints, of which there are only three, exclusive of the head. The latter is surmounted by two rows of blunted hooklets, fifteen to twenty-five in each row. This parasite attacks man, the domestic animals such as the horse, cow, sheep, goat, or hog, and some wild animals; and has even been seen in the turkey (Von Siebold). When the human being has been selected as the site for the larval form—the ecchinococcus—we find a sac of varying size, which contains the little animal. So small may this object be, that it is barely recognized by the unaided eye; or, on the other hand, so large that it forms a very considerable tumor, altering the form of the body, and clearly appreciable by palpation. On the inner surface of the cyst are numbers of little elevations, which enclose small capsules—the so-called brood-capsules—from the walls of which the tape-worm heads depend. Being endowed with a capacity for active movement, the little animals elongate or contract upon their pedicles, and so immerse themselves at will in the fluid of the general cyst.

Each head is covered with four discs or suckers in addition to the hooks. Sometimes these ecchinococci become separated from their base and float about at large. Since the parasite in this condition has a very feeble power of locomotion, it is difficult to believe that it forces its way through the tissues in reaching its usual haunt, the human liver. Authorities regard it as most probable that the avenues of entrance are either the bile ducts or portal vein and its tributaries. We should not forget that Küchenmeister has intimated that man may also harbor the mature form of the parasite, *i. e.*, the tænia ecchinococcus and the development of the sacs is then to be explained by self-infection. This view has received some recent confirmation.

The word "hydatids" has sometimes been applied to this disease, but it is likely to cause confusion, because it is associated often with an altogether different affection, viz., uterine hydatids, a cystic metamorphosis of the chorion, which does not even bear an external resemblance to the ecchinococcal disease produced by the parasite. Genuine instances of the latter are rarely found in the uterus proper, though it is not uncommon to find them in the sub-peritoneal tissue. Graily Hewitt suggests, with some propriety, that if the uterine wall is really involved, the lesion may be secondary, as indeed it is not uncommon for the liver tumor to soften, discharge into the peritoneum, and finally be expelled from the body through some suppurating tract, just like any foreign body in a similar situation. In fine, uterine "hydatids" are little pellucid sacs caused by a cystic change in the villosities of the chorion; ecchinoccocal "hydatids" are multilocular sacs of considerable firmness, which either communicate with the general cyst cavity, or are enclosed within one another, and are caused by the presence of animal parasites.

The word "acephalocyst" has been applied to the sacs where no heads could be found, but it is now thought best to abandon it, because the absence of a head does not denote the absence of the animal, but rather its infrequency, sparseness, or imperfect development, as in a case mentioned by Heller, in which there were numberless sacs in the liver and lungs of a cow, and yet very few scolices or heads. Every organ of the body may be affected, but the liver is the chief haunt.

The so-called multilocular ecchinococcus is now regarded as a peculiar form of the same disease. When found in the human liver—its usual seat—there is a hard mass which supplants the liver tissue proper, and in some places increases so as to become a tumor of gigantic size. It consists of a fibrous tissue of very firm texture, interspersed with a network of intercommunicating spaces, which, from their arrangement, were supposed by Virchow to indicate that the lymphatics were the original seat of the lesion. Friedreich, on the other hand, has intimated that the blood-vessels or gall-ducts are first attacked.

Neither scolices nor hooklets are found with any regularity, and the tissue is prone to break down and form an abscess.

Thus far, according to Heller, we have had reports of the multilocular ecchinococcus in only thirty-nine cases (cattle four, man thirty-five.) Of these, twenty-seven have been more or less accurately observed. Fourteen occurred in Switzerland, eight in Bavaria (the southern part, mostly), seven in Wurtemberg, three in Austria, one each in Baden and Dorpat, and one in an unknown part of the country.

The least age of the human cases was nineteen; the greatest, sixty-eight. In twenty-one of the cases either scolices or hooklets were found; in eight they were not found.

In the human species the supra-renal capsule was involved but once; the liver twenty-five times. Four times the multilocular ecchinococcus was found in the lungs. The parasites were usually not confined to their first seat, but were scattered over other parts of the liver, including the lobulus spigelii, lobus quadratus, the portal lymphatic glands, and the peritoneum. Perforations of the vena porta, hepatic veins, and biliary passages occurred.

The subjoined table, by Finsen, gives a record of 255 cases that were observed in Iceland, where the disease is very rife, owing to the very great numbers of dogs. With each organ is given the number of times it was involved: Liver, 176; kidney, 3; spleen, 2; peritoneal cavity (original site uncertain), 54; lungs, 7; head, 4; mamma, 1; other portions of the surface of the body, 8.

Of thirty-four cases, with doubtful primary lesion, the liver was certainly affected in the majority. Of 189 cases, fifteen were in children under ten years of age; thirty-five between ten and twenty; forty-nine between twenty and thirty; twenty-three between thirty and forty; twenty-seven between forty and fifty; twenty-one between fifty and sixty; fourteen from sixty to over eighty; five uncertain. The youngest case was four years old; the oldest, eighty-seven.

It is said that in some of Finsen's patients, the disease had exhibited itself at the early age of two years. Among the more recent cases is one of sudden paralysis, reported by Liouville and Strauss (*Progres. Med., 1875, No. 4*). The cause was found to be compression of the medulla by an ecchinococcus, which had effected an entrance into the spinal canal from without, at the level of the tenth spinal vertebra.

The symptoms in the human species exhibit many variations, which need particular description. The following summary is given by Heller: At first there are gastric disturbances, with occasional pains in the epigastrium, or right hypochondrium; later, a sense of fullness and weight in the belly; as a rule, jaundice appears early; at first it is slight, but soon increases, and is apt to reach great intensity. Digestion is generally disturbed at an early period; constipation alternates with diarrhœa; the passages usually have a clay color, though they sometimes appear normal. Soon the patient observes that there is a distinct swelling of the belly, increasing gradually and almost imperceptibly. Examination reveals a decided and often very considerable enlargement of the liver; it is of varying consistence, though the surface is smooth. When the affected portions are accessible to the touch, they appear firm and even, or irregularly tuberculated; sometimes, though seldom, hard nodules are to be felt. The spleen is almost always considerably enlarged, and sometimes not easily demarcated from the liver. No important disturbances are to be observed in the respiratory organs and nervous system. Pain in the liver is sometimes constantly present, sometimes periodically increased. Occasionally it is absent and is only to be detected by pressure. Frequently there is a tendency to hæmorrhages; in the last stages there are dropsical swellings. Fever does not usually ensue until the end of life approaches, and is often superinduced by other accessory inflammatory processes. Death is usually the result of exhaustion, but sometimes is caused by other complications, such as cerebral hæmorrhage, pericarditis or peritonitis.

The diagnosis is very difficult during lifetime, since the symptoms, as may be gathered from the preceding statements, offer nothing distinctive of this disease. In making a diagnosis, the ordinary ecchinococcus affection and cancer of the liver are to be differentiated. In the former the enlargement of the spleen is, as a rule, absent, while in the multilocular form it is almost always present; in the former jaundice is very rare, in the latter almost the rule; fluctuation in the latter is only exceptional, and an exploratory puncture would make the distinction, since the simple form would yield a fluid of the material that has previously been described, while the contents of the gangrenous cavity, which forms in the multilocular variety, is always more or less stained with bile. In distinguishing this affection from cancer of the liver, the course of the disease is to be specially noted. Cancer of the liver always has a more speedy course, while the ecchinococcus has a long continuance. Cancer of the liver that has progressed some weeks exhibits alterations in form and size; the general disturbances of nutrition are also far less frequent; so, too, there is seldom enlargement of the spleen; on the contrary, this is usually small and atrophic; it is only in cases that run a very acute course that they are swollen; in these the ecchinococcus disease is excluded on account of the rapid course. In cancer of the liver there is always a possibility of feeling the cancerous nodules by the projection of the liver below the free borders of the ribs. Abscess of the liver, waxy liver, and hepatic syphilis are not difficult of diagnosis; and it may also be said that the rarer hypertrophic form of cirrhosis of the liver may be excluded without much difficulty. It is very questionable whether an examination of the intestinal discharges, with reference to fragments of the ecchinococcus, will establish a diagnosis.

The prognosis is utterly unsatisfactory. All the cases that have thus far been known have terminated fatally. Treatment, except in the cases of Griesinger and Jürgensen, has been purely symptomatic, since it is absolutely hopeless. Prougeansky wisely recommends operative procedures, with the faint hope that a cure may be reached in this way.

THOMAS E. SATTERTHWAITE, M. D.

The thirty-five cases spoken of by Heller may be studied by consulting the following thirty five references:

1. BAUER, Würtemb. Corresp-Blatt. 42. No. 26. 1872......2. Böttcher, Virch. Arch. 15. S 352. 1858...... 3. Bosch, Dissert. inaug., Tübingen, 1868...... 4. Buhl, Illustr. med. Ztg. 1 S. 102. 1852...... 5 Buhl, Zeitschr. f. ration. Med. N. F. 4. S. 356. 1854. (Verhandlungen d. Würzb. med. Gesellsch. 6. S. 428. 1855.)......6. Carrière, De la tumeur hydat. alvéol. Paris 1868. (Féréol, L'Union méd. 1867. No. 114)...... 7. Ducelier, Bullet. de la soc. mèd. de la Suisse romande. 1868. p. 193. (Etude clin. sur la tumeur à ēchin. multiloc. Paris. 1868.)......8. Erismann, Diss. inaug. Züric. 1864......9. Friedreich, Virch. Arch 33. S. 16. 1865......10. Griesinger, Archiv. der Heilkunde 1. S. 547. 1860....... 11. Haffter, ibid. 16. S. 362. 1875...... 12. Heschl. Prag. Vierteljahrschr. 50. S. 36. 1856. II...... 13. Heschl, Oesterr. Zeitschr. f. pract. Heilkunde. VII. 5. 1861. (Schmidt's Jahrb. 116. S. 188. 1862)...... 14. Huber, Deutsches Archiv. f. klin. Med. 1 S 539. 1865 ...... 15. Huber, ibid. 4. S. 613 u. 5. S. 139. 1869......16. u. 17. Kappeler, Arch. d. Heilkunde 10. S. 400. 1869......18. Klebs, Handbuch d. patholog Anatomie. S. 517. 1869...... 19 Leuckart, Parasiten. I. S 372 ......20. Luschka, Virch Arch. 4. S 400. 1852......21. Miller, Dissert. naug. Tübingen 1874...... 22. u. 23. Morin. Dissert. inaug. Bern 1876...... 24. und.. 25. Ott, Berliner klin. Wochenschr. 1867. S. 299. 311. 327. 339...... 26. Prévost, Bullet. de la soc. mèd. de la Suisse romande. 1875. p. 5...... 27. 28 29. Prougeanski, Dissert. inaug Zü ich. 1873 ......30. Scheuthauer, Oesterr. med. Jahrb. 14. S. 17. 1867. (I. Fall)...... 31. Schiess, Virch. Arch 14. S. 371. 1858......32. Virchow, Verh. d. Würzb. med. Ges 6. S. 84. 1856. ...... 33 Virchow, Arch. 11. S. 80 1857. ...... 34. Zeller, Dissert. inaug. Tübingen 1854 ...... 35. Zenker (unpublished) To these may be added the following probable cases. Dittrich. Prag Vierteljahrschr. 1848. 3. S. 118. Meyer. Diss. inaug. Zürich. 1854. (several cases.) The following four occurred in cattle: 1. Huber. Jahrb. d. naturforsch. Gesellsch zu Augsburg, 1861. (Mch. Arch. 54 S. 269. 1872.) 2–4 Bollinger. Deutsche Zeitschr. f. Thier. med. 2. S. 109. 1875. The cases cited by Ott and reported by Coulson, Baudelocque. and Fricke, are not examples of multilocular ecchinococcus: the same may be said of Scheuthauer's cases, and those of Goodsir and Gairdner, given by Carriere.(Heller). The following references way also be consulted:

DÜTSCH. Dissertat inaug. Kiel. 1869.

KUSSMAUL. Berliner Klin. wochenschrift. 1869. S. 545.

MUNK. Virchow's Arch. 63. S. 560. 1875.

HELLER Ziemssen's Handbuch. III. Band. 2 Auflage. 1876.

GALLIOT. Bull. gen. de Ther. Paris. 1879. xcvii 97–108.

KUCHENMEISTER and Zurn. Die Parasiten des menschen. 2. Aufl. 2 Lfg. Leipsig. 1879 1. Abtheil.

COBBOLD. Entozoa, London. 1879.

MALASSEZ. Gaz. med. de Paris. 24 Arvil. 1880.

